# Facile Synthesis of Ultralong and Thin Copper Nanowires and Its Application to High-Performance Flexible Transparent Conductive Electrodes

**DOI:** 10.1186/s11671-018-2486-5

**Published:** 2018-03-07

**Authors:** Yaxiong Wang, Ping Liu, Baoqing Zeng, Liming Liu, Jianjun Yang

**Affiliations:** 1 School of Physical Electronics, University of Electronic Science and Technology of China, Chengdu, 610054 China; 2 College of Electron and Information Engineering, University of Electronic Science and Technology of China Zhongshan Institute, Zhongshan, 528402 China

**Keywords:** Copper nanowire, Transparent conductive electrode, Hydrothermal synthesis

## Abstract

A hydrothermal method for synthesizing ultralong and thin copper nanowires (CuNWs) with average diameter of 35 nm and average length of 100 *μ*m is demonstrated in this paper. The concerning raw materials include copric (II) chloride dihydrate (CuCl_2_·2H_2_O), octadecylamine (ODA), and ascorbic acid, which are all very cheap and nontoxic. The effect of different reaction time and different molar ratios to the reaction products were researched. The CuNWs prepared by the hydrothermal method were applied to fabricate CuNW transparent conductive electrode (TCE), which exhibited excellent conductivity-transmittance performance with low sheet resistance of 26.23 $\Omega /\square $ and high transparency at 550 nm of 89.06% (excluding Polyethylene terephthalate (PET) substrate). The electrode fabrication process was carried out at room temperature, and there was no need for post-treatment. In order to decrease roughness and protect CuNW TCEs against being oxidized, we fabricated CuNW/poly(methyl methacrylate) (PMMA) hybrid TCEs (HTCEs) using PMMA solution. The CuNW/PMMA HTCEs exhibited low surface roughness and chemical stability as compared with CuNW TCEs.

## Background

Transparent conductive electrodes (TCEs) are extremely important parts in many optoelectronic devices including organic light-emitting diodes (OLEDs), solar cells, liquid crystal displays, Flat-panel displays, sensors and so on [1–7]. Indium tin oxide (ITO) is one of the most commonly used TCEs in industry, which possesses low resistivity ($\sim \thinspace \!\!10-30 \Omega /\square $) at high optical transparency (90%) [8]. It is a great pity that indium is a rare metallic element and its’ abundance in the earth’s crust is very low, which leads to the price of ITO becoming more and more expensive [8–10].

Hence, researchers have done a lot of attempts to develop some new materials to partially replace ITO. These candidates should possess low cost, high conductivity, high transmittancy, and excellent flexibility and can be deposited at low temperatures. Among those candidates, metallic nanowires are especially promising. Recent studies have reported the use of silver nanowires (AgNWs); transparent electrodes based on AgNWs have been reported to compete well with ITO [11–19]. However, silver is a precious metal, and its expensive price should not be ignored. As a result of the increasing demand for low-cost metallic nanowires, copper has received considerable attention as an interesting alternative to silver. Copper is almost as conductive as silver, because bulk resistivity of copper is 1.67 *nΩ*·*m*, while that of silver is 1.59*nΩ*·*m* [20]. Moreover, copper is much more abundant and far less expensive than silver and ITO. Based on these facts, more and more attention is paid to copper nanowires (CuNWs).

Thus, various methods for preparing CuNWs have been studied, such as chemical vapor deposition, electrochemical deposition, template, and membrane processes [21–26]. However, these methods involve several complex processes and require toxic chemical reagents or valuable catalyst. Probably, hydrothermal methods seem to be one simple way for the production of CuNWs. Zhang et al. prepared CuNWs (or copper nanorods) with diameter being about 50 nm and length reaching up to > 10 *μ*m by means of hydrothermal synthesis with ascorbic acid as reducing agent and polyvinyl pyrrolidone (PVP) as capping agent at relatively low temperature [27]. Wang et al. prepared ultralong CuNWs with a uniform diameter of about 800–1000 nm and a typical length of several tens of micrometers by applying ascorbic acid as reductant and capping agent [28]. Using octadecylamine (ODA) as both a soft reducing agent and an adsorption agent, Shi et al. obtained ultralong CuNWs with length up to several millimeters and diameters of 30–100 nm [29]. Melinda Mohl and co-workers applied the copper chloride and glucose in the presence of hexadecylamine (HDA), successfully prepared some long CuNWs with a uniform diameter of 64 ± 8 nm and length of a few micrometers [30]. Aziz et al. elaborated a simple hydrothermal method to produce CuNWs with length of twenties micrometers by using HDA and potassium bromide as capping agent [31]. Kim et al. reported a seed-mediated synthetic strategy for CuNW production, and some typical scanning electron microscopy (SEM) images on CuNWs showed that average diameter was 21.9 ± 3.8 nm, and the maximum length was 77.1 *μ*m [32].

Compared with CuNWs, fabrication on CuNW TCEs has been scarcely studied, since instability and the feature of being easily oxidized often lead to CuNW films to be non conductive. Wiley and coworkers have achieved good results in the preparation of CuNWs and CuNW TCEs. In 2010, it was the first time that they had prepared CuNW TCEs on flexible substrate with a sheet resistance of 30 $\Omega /\square $ at specular transmittance of 85% by means of Meyer rod coating [33]. In 2014, They improved the way to produce CuNWs and then made CuNW TCEs with a transmittance > 95% at a sheet resistance < 100 $\Omega /\square $ [34]. Simonato’s group treated CuNWs with glacial acetic acid to fabricate flexible CuNW TCEs exhibiting a sheet resistance of 55 $\Omega /\square $ at 94% transmittance (*λ*=550 nm) by means of vacuum filtration [20]. Chu et al. prepared CuNW TCEs with 52.7 $\Omega /\square $ at *T*=90% (*λ*=400–700 nm) using a spray coating, which were not conductive before annealing in a furnace for 2 h in an atmosphere of 75% argon and 25% hydrogen [35]. However, despite these efforts, CuNW-based TCEs still present a number of limitations that prevent their widespread use. One problem is their high surface roughness when deposited on bare substrates and another problem is that CuNWs have low oxidation potential and chemical stability.

One way to improve the performance of nanowire films is to use nanowires with higher aspect ratios [34]. Short and coarse CuNWs are not suitable for the preparation of TCEs. For example, CuNWs with diameters about 50 nm and length about 10 *μ*m proposed in Ref. [27] are too short to prepare high-quality TCEs. It is worth noting that too long CuNWs are also not suitable for the preparation of TCEs, because it is too easy for them to get together and can not be well-dispersed. For example, length of CuNWs proposed in [29] was up to several millimeters, and many of CuNWs bundle together [29]. Moderate lengths and diameters of CuNWs are very important for high-quality CuNW TCEs. In this paper, a simple hydrothermal approach for the synthesis of thin, well-dispersed, and ultralong CuNWs with average diameter of 35 nm and average length of 100 *μ*m is reported, where ODA, ascorbic acid, and copric (II) chloride dihydrate (CuCl_2_·2H_2_O) are involved. CuCl_2_·2H_2_O provides copper source, ascorbic acid is used as reducing agent, and ODA is selected as capping agent. The lengths and diameters of CuNWs are affected by the reaction time, molar ratio of the three drugs, and we will reveal the effects of these factors. The ultralong and thin CuNWs were used to fabricate CuNW TCEs at room temperature. The sheet resistance of the CuNW TCE was as low as 26.23 $\Omega /\square $ at 89.06% transmittance (*λ*=550 nm). In order to reduce roughness of CuNW TCEs and prevent CuNWs from being oxidized, poly(methyl methacrylate) (PMMA) was coated on surface of CuNW TCEs to prepare CuNW/PMMA hybrid TCEs (HTCEs), and the effects of PMMA on transmittance and roughness of CuNW TCEs are demonstrated.

## Experimental

### Synthesis of CuNWs

In a typical process for synthesizing CuNWs, 140-mg ascorbic acid (C_6_*H*_8_*O*_6_, Aladdin) and 270-mg CuCl_2_·2H_2_O (Aladdin) were added to 282 ml of ODA (26.3 mmol L^−1^) aqueous solution. Molar concentrations of ascorbic acid and CuCl_2_·2H_2_O are 2.8 mmol L^−1^ and 5.6 mmol L^−1^, respectively. The mixed solution turned to homogeneous suspension after 60 min of normal temperature sealing stirring. Subsequently, the obtained suspension was transferred into a Teflon-lined autoclave and sealed for 20 h at 120 °C. The reactor was then cooled down to room temperature naturally. The excess chemicals were removed by washing with deionized water and ethanol. The final product was kept in 130-ml glacial acetic acid (Aladdin) to avoid oxidation of CuNWs.

### Fabrication of CuNW TCEs and CuNW/PMMA HTCEs

CuNW TCEs were fabricated on Polyethylene terephthalate (PET) substrates (188 *μ*m thickness). A slight amount of glacial acetic acid solution containing CuNWs was diluted by 500-ml deionized water. TCEs were formed at room temperature by filtration of a dispersion of CuNWs on a mixed cellulose ester (MCE) filter membrane (0.45 *μ*m). The deposited film was then transferred to the PET substrate by applying uniform pressure. The MCE filter membrane was peeled off to keep the CuNW network on the PET substrate. 100 ul PMMA solution(20 mg/ml) was coated onto surface of CuNW TCEs using a spin coater at 800 rpm for 5 s and 2500 rpm for 30 s. CuNW/PMMA HTCEs were naturally dried without thermal sintering.

### Structural, Optical, and Electrical Characterization

The morphology and dimensions of the synthesized CuNWs were investigated by SEM (JSM-7500F, JEOL) and transmission electron microscopy (TEM) (FEI-TECNAL G20). Surface morphology images of the CuNWs were analyzed by an optical microscope (BX51M, OLYMPUS). The transmittances of CuNW TCE and CuNW/PMMA HTCE were determined by a UV spectrophotometer (GZ502A, Shanghai shine Photoelectric Technology Co., Ltd.). Roughness of CuNW TCEs and CuNW/PMMA HTCEs was determined by an atomic force microscope (AFM) (Dimension Edge, BRUKER). The powder X-ray diffraction (XRD) patterns of CuNWs were performed by XRD analysis (Bruker, BRUKER OPTICS).

**Fig. 1:**
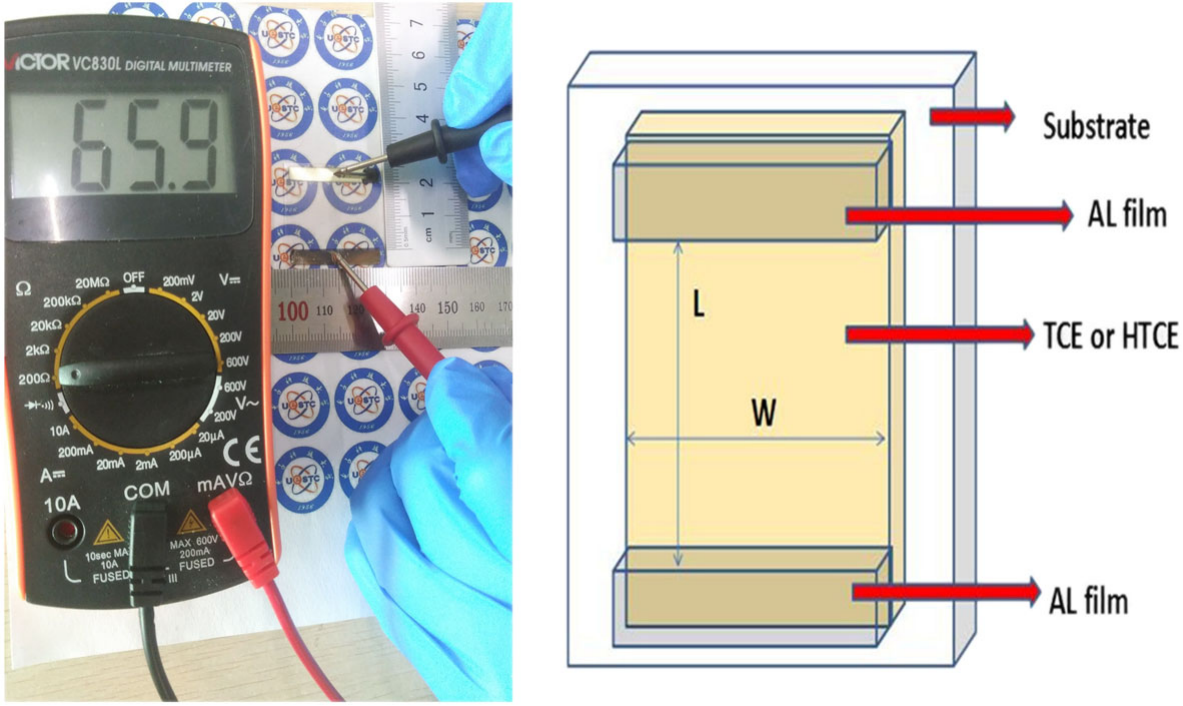
Photographic image and schematic illustration testing resistance process. Left is photographic image of testing resistance process, and right shows schematic illustration of length and width of a TCE

Two sheets of aluminum (Al) film were deposited at both ends of a CuNW/PMMA HTCE or a CuNW TCE, as shown in Fig. 1. The distance between the two inner sides of the aluminum films is marked as length *L*, and the distance between the other two sides of the TCE is marked as width *W*. Relationship of resistance *R* of a film and its sheet resistance *R*_*s*_ is constrainted by the formula as follows [36]:

1 $$\begin{array}{@{}rcl@{}} R_{s}=R\frac{W}{L} \end{array} $$

Resistance of CuNW TCE and CuNW/PMMA HTCE was measured by a multimeter and the sheet corresponding resistances were concluded from the resistances with the help of Formula (1). For example, the resistance of a CuNW/PMMA HTCE is 65.9 *Ω*, as shown in Fig. 1, *L* is 19.2 mm and *W* is 27.6 mm, respectively. Then, one can conclude that the sheet resistance of the CuNW/PMMA HTCE in Fig. 1 is 94.7 $\Omega /\square $.

## Results and Discussion

### Synthesis of CuNWs

In this work, well-dispersed CuNWs with an average diameter of 35 nm, an average length of 100 *μ*m, and aspect ratio about 2857 were synthesized by the reduction of CuCl_2_·2H_2_O with ascorbic acid through a hydrothermal reduction process. ODA acted as capping agent in the process of the growth of CuNWs. The three materials are all very cheap, so the cost of preparing CuNWs is very low. Different reaction time and different molar ratios will lead to different products, and we will discuss these factors in the following subsections.

#### (A) Characterization of CuNWs

**Fig. 2:**
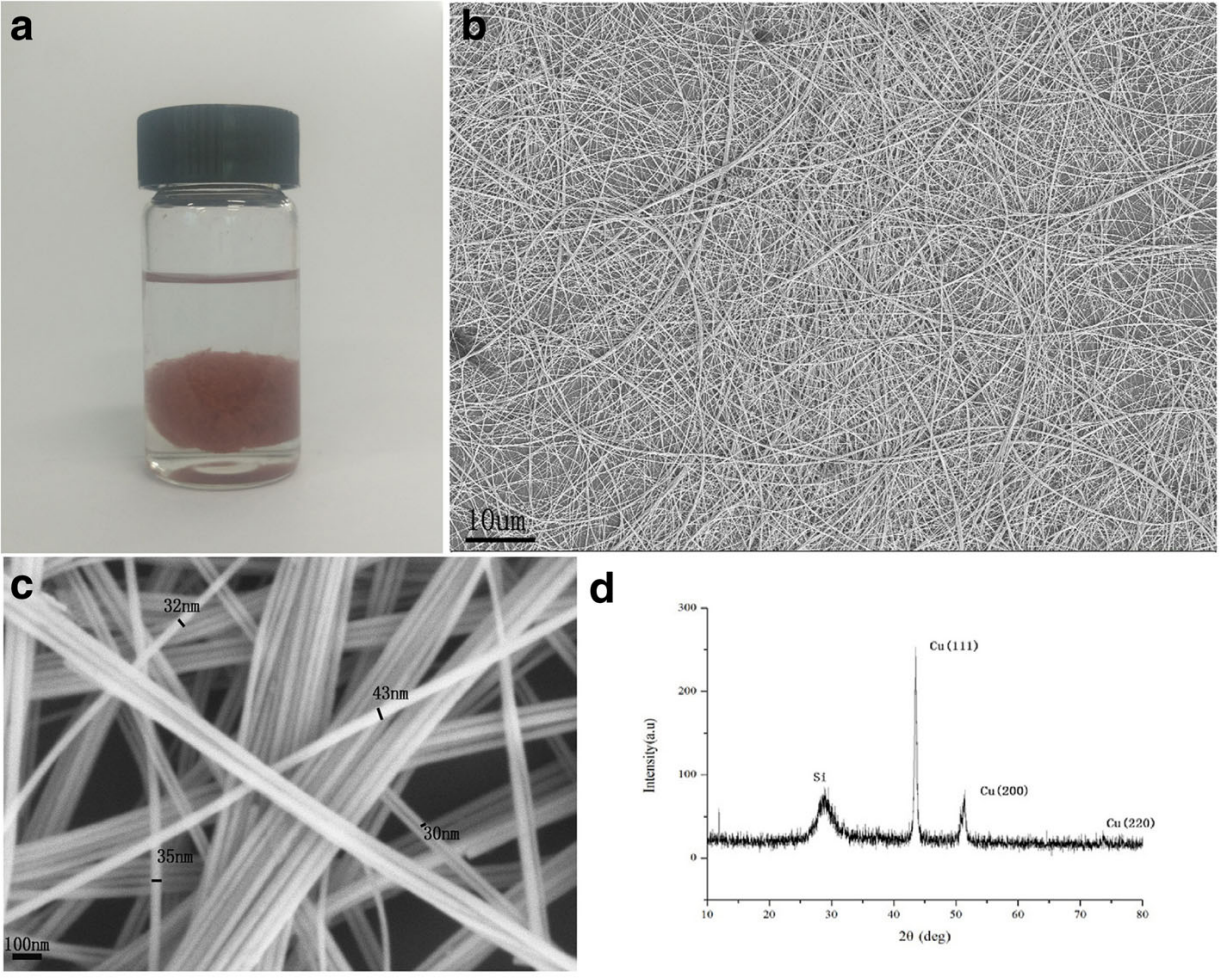
Structure of CuNWs. **a** The CuNWs as-prepared in acetate. **b** SEM image of CuNWs from a general view. **c** SEM image of CuNWs from a detailed view. **d** Powder XRD pattern of the CuNWs drop-casted on a silicon substrate

The CuNWs were prepared by the method in the “Synthesis of CuNWs” subsection of the “Experimental” section. The photograph of CuNWs obtained is shown in Fig. 2a. The morphology and dimension of the corresponding CuNWs are shown by SEM in Fig. 2b, c. It is shown in Fig. 2b, c that the final product is composed of a large quantity of CuNWs with average diameter of 35 nm and average length of 100 *μ*m, thus featuring an aspect ratio about 2857. Long NWs with a high aspect ratio are required in order to achieve high electrical conductivity and transmittance in the NWs-based web structures, because they enable fully inter-networked, highly conductive pathways even with low nanowire density [37, 38]. Consequently, the method mentioned above is applicable for the production of uniform CuNWs. These thin and uniform CuNWs make it possible to prepare high-performance CuNW TCEs. Figure 2d depicts the XRD pattern of CuNWs drop-casted on a silicon substrate. CuNWs are identified on the basis of the three distinguishable diffraction peaks at 43.316, 50.448, and 74.124, corresponding to {1 1 1}, {2 0 0}, and {2 2 0} crystal planes of face-centered cubic copper, respectively. The intensity of {1 1 1} crystal planes of face-centered cubic copper higher than those of the others indicates the enrichment of {1 1 1} crystal planes of the CuNWs. The result also indicates that copper atoms firstly deposited on {1 1 1} facets, led to one-dimensional (1-D) CuNWs. No signal from impurities such as CuO and Cu_2_O was observed in the XRD pattern, which indicates pure CuNWs were obtained.

**Fig. 3:**
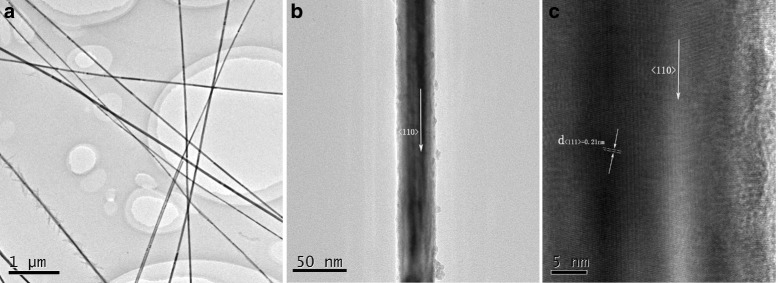
TEM images and HRTEM image of CuNWs. **a**, **b** TEM images of CuNWs. **c** HRTEM image of a CuNW. The lattice spacing was observed to be 0.21 nm

Figure 3a, b shows TEM images of ultra-long CuNWs with diameters about 35 nm. We can observe that the surfaces of the copper nanowires are very smooth. Figure 3c proposes high-resolution TEM (HRTEM) image of a CuNW. The lattice spacing was observed to be 0.21 nm, which corresponds to the {1 1 1} plane of face center cubic copper.

#### (B) Time-Dependent Analysis

**Fig. 4:**
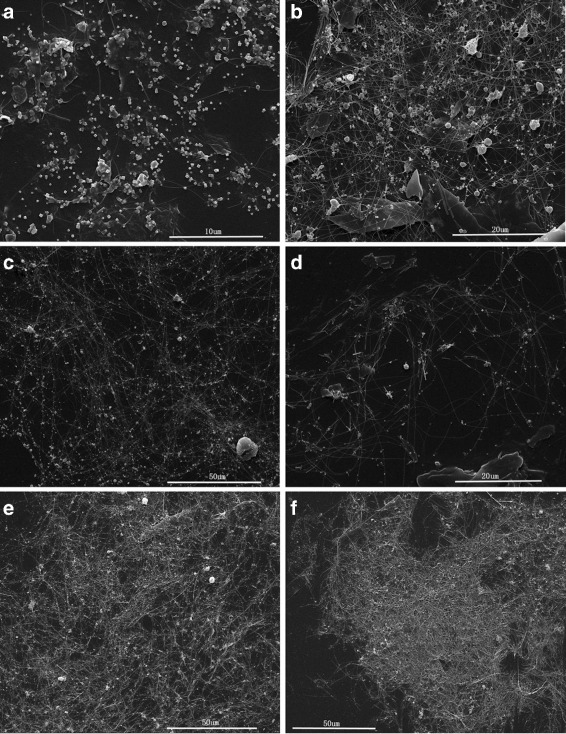
SEM images of samples at different reaction time of hydrothermal treatment. The different reaction time of hydrothermal treatment are **a** 1 h, **b** 2 h, **c** 6 h, **d** 14 h, **e** 20 h, and **f** 40 h

In order to observe the growth process of the CuNWs, the products were investigated by SEM. Products obtained after 1- to 40-h hydrothermal treatment at 120 °C exhibited different morphologies and length. Morphology of CuNWs with different reaction time are studied by SEM. SEM images of samples at different stages of hydrothermal treatment are shown in Fig. 4.

From Fig. 4a, one can see that the product is mostly composed of copper nanocubes and almost no CuNWs is produced when the reaction time is 1 h, which indicates an isotropic growth dominated system. The reason is that the capping agent was insufficient to cover all newly formed {1 0 0} facets on copper nanocrystals in this stage. Since the growth mechanism of CuNWs is not yet fully understood, so we speculate that only a small fraction of the ODA is vaporized and dissolved under high pressure at the initial stage. Therefore, there is no enough capping agent to ensure the anisotropic growth of the crystal.

As time goes on, more and more ODA are gasified to produce sufficient capping agents, and then 1D growth on these nanocrystal seeds in aqueous leads to the formation of CuNWs. As shown in Fig. 4b–d, more and more raw materials turn to products and more and more nanocrystal seeds convert into CuNWs when time goes from 2 to 14 h. Meanwhile, we can see that the lengths of the CuNWs are becoming longer and longer in the range of 2 to 14 h, and the average length of CuNWs is about 25, 60, and 80 *μ*m in Fig. 4b–d, respectively. The CuNWs are very thin and their average diameter is only about 35 nm. When the CuNWs are longer than 100 *μ*m, they become to be easily broken, so their lengths no longer increase. Figure 4e, f indicates that the lengths and diameters of the CuNWs do not change significantly within 20 to 40 h.

#### (C) Amount of Ascorbic Acid

Ascorbic acid was selected as reducing agent in the synthesis of CuNWs. To investigate the influence of the amount of ascorbic acid on the morphology of the CuNWs, the amounts of deionized water, ODA, and CuCl_2_·2H_2_O were fixed and the amount of ascorbic acid was changed. After 20 h of heating in a sealed Teflon-lined autoclave at 120 °C, the obtained solution was washed with deionized water and ethanol. The final products were observed with the help of an optical microscope.

**Fig. 5:**
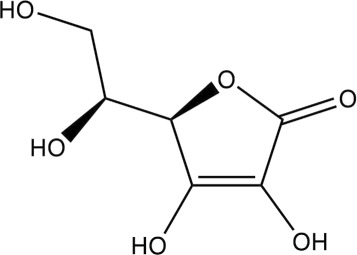
Chemical structural formula of ascorbic acid. There are four hydroxyls (-OH) in one ascorbic acid molecule, which act as the functional group in this reduction reaction

**Fig. 6:**
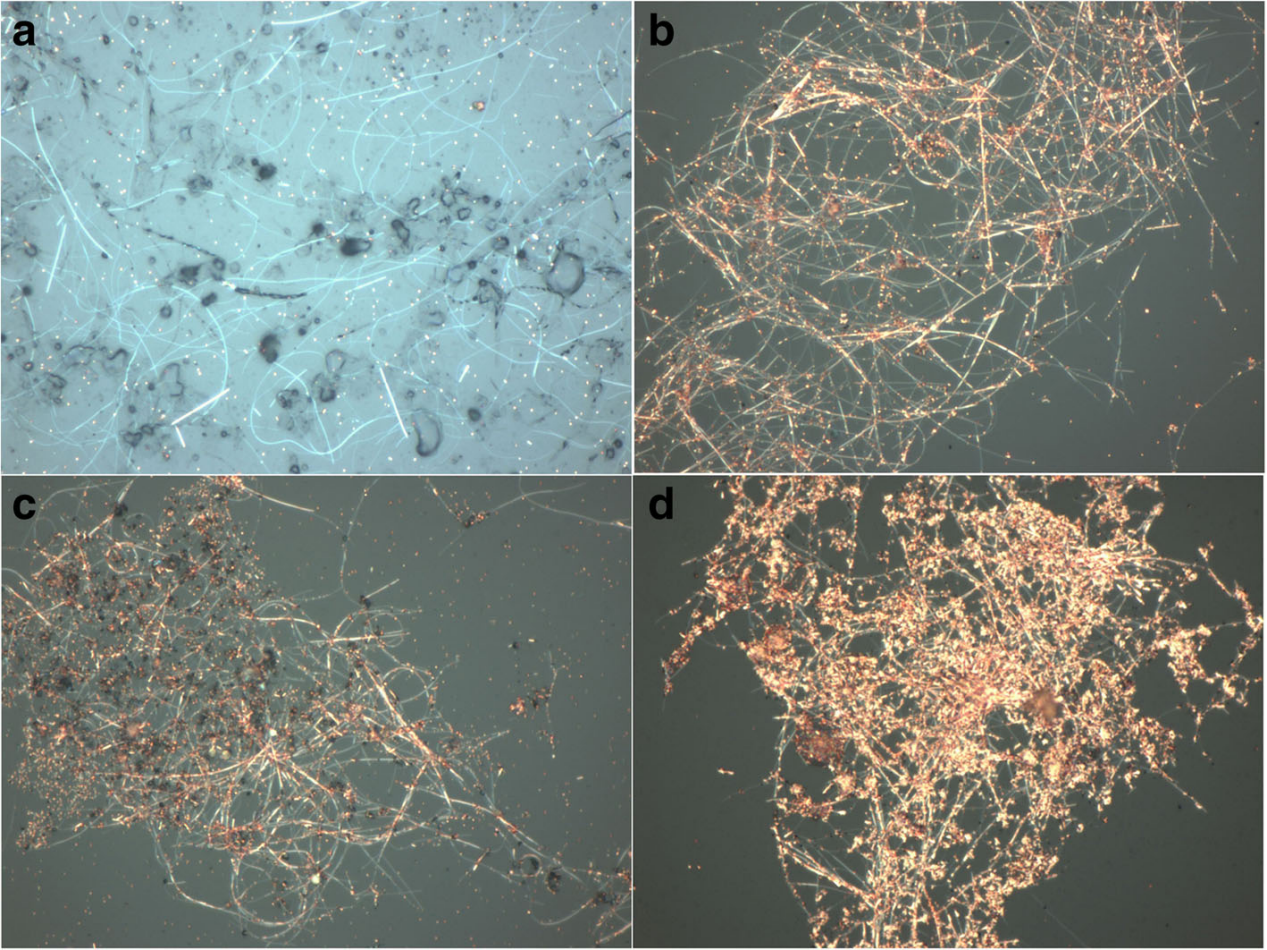
Optical microscope images of CuNWs synthesized with different molar ratios of ascorbic acid and CuCl_2_·2H_2_O. The different molar ratios of ascorbic acid and CuCl_2_·2H_2_O are **a** 0.2:1, **b** 0.5:1, **c** 1:1 and **d** 2:1

Chemical structural formula of ascorbic acid can be written as shown in Fig. 5. There are four hydroxyls (-OH) in one ascorbic acid molecule, which act as the functional group in this reduction reaction, and there is one Cu^2+^ in one CuCl_2_·2H_2_O molecule, so one can assume that optimum molar ratio of ascorbic acid and CuCl_2_·2H_2_O is 0.5:1. Figure 6 demonstrates the optical microscope images of the final products with different molar ratios of ascorbic acid and CuCl_2_·2H_2_O. When the molar ratio is 0.5:1, the largest amount of CuNWs is produced (Fig. 6b) and the amount of copper nanoparticles (CuNPs) is relatively much smaller. Upon increasing the molar ratio to 1:1 or 2:1, more and more CuNPs appear in the optical microscope images (Fig. 6c, d). At the molar ratio of 2:1, the product contains a large number of CuNPs and any CuNWs are hardly formed. This is probably because that CuNWs grow from multi-twinned seeds. However, multi-twinned seeds are unstable at initial stages if their {1 0 0} facets are not well capped. the crystal growth on the {1 0 0} facets could rapidly develop multi-twinned seeds into single-crystal seeds, which only produce CuNPs. When the molar ratio of reducing agent is much larger than the appropriate value, a large number of multi-twinned seeds will appear at the beginning stage. At the same time, the ODA is not heavily vaporized, and diffusing in the aqueous, resulting in the lack of capping agent. In the case of the ratio is 0.2:1 in Fig. 6a, the solution is very viscous and contain a large amount of ODA, which makes it very difficult to separate the CuNWs from the solution. Small amounts of CuNPs and CuNWs are produced in Fig. 6a, and we can assume that in this case only part of copper are reduced since the amount of reducing agent is less than normal.

### Fabrication of CuNW TCE

The vacuum filtration transfer method is a simple and scalable method to make TCEs. By means of vacuum filtration transfer, the solutions containing NWs can be adequately diluted and be dispered as individual wires, so the prepared TCEs possess good conductivity. Meanwhile, some reagent can be filtered out, which is helpful to improve electrical conductivity of the TCEs. In this work, the glacial acetic acid solution containing CuNWs is diluted by deionized water and then filtered on a MCE filter membrane. During the process, the residual organic materials and copper oxides can be removed, which helps to improve electrical conductivity of the CuNW TCEs.

**Fig. 7:**
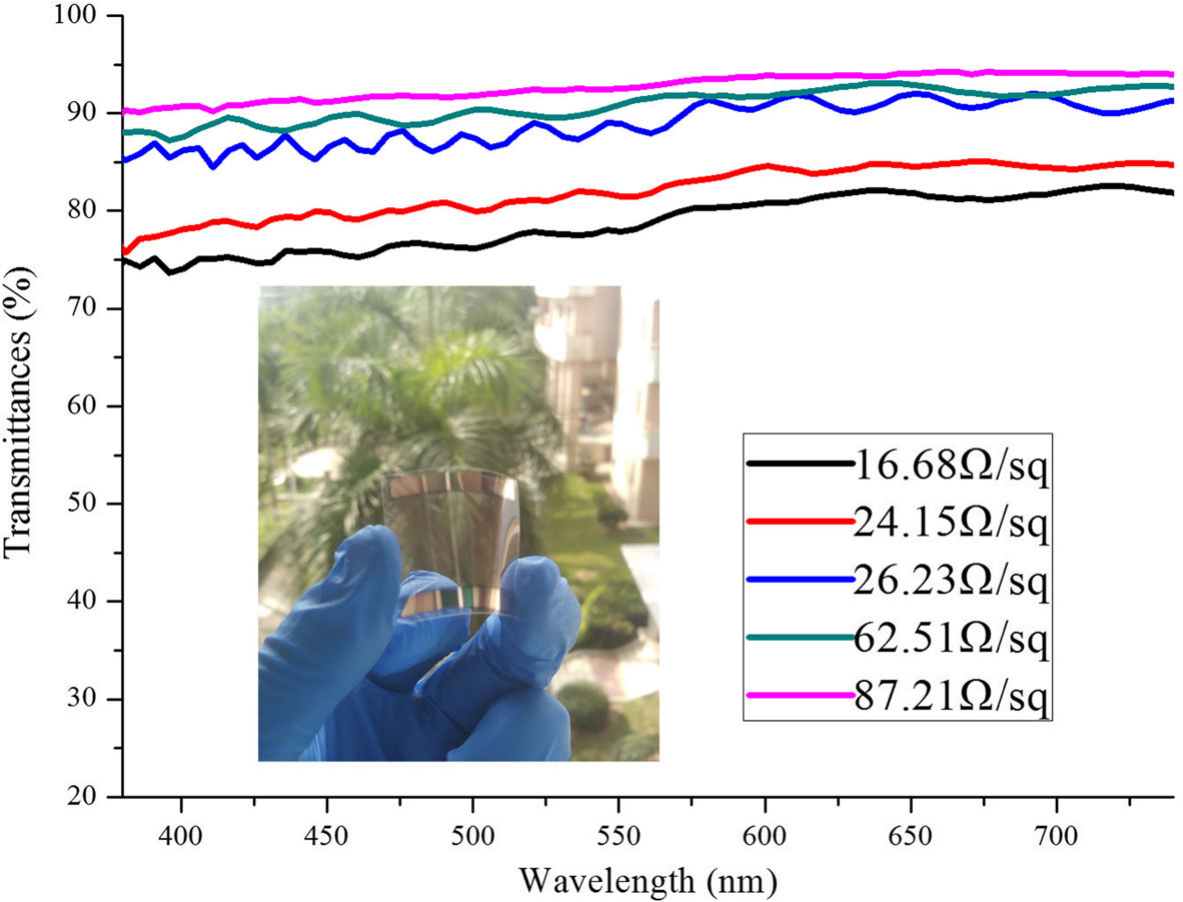
Transmittance spectra (366–741 nm) of the CuNW TCEs with various sheet resistances. Sheet resistances and transmittance of the five samples at 550 nm are listed in Table 1. Photographic image of a CuNW TCE is demonstrated, whose transmittance at 550 nm is 89.06% on condition that effect of PET is excluded

The transmittance of a TCE depends on the amount of CuNWs deposited on the substrates, and it will drastically decrease when the sheet resistance of the TCE decreases. High conductivity and high transmittancy of the CuNW TCEs are mainly attributed to the morphology of CuNWs such as long length, small diameter, absence of NPs and any other residual organic matters [39–41]. In this work, we select the reaction time as 20 h and molar ratio of ascorbic acid and CuCl_2_·2H_2_O as 0.5:1. Table 1 lists sheet resistances and transmittances (excluding PET substrate) at 550 nm for five CuNW TCEs. The fact that higher transmittances are related to lower sheet resistances is in line with the existing observations. Transmittances at wider range (*λ*=366–741 nm) for the five samples are proposed in Fig. 7. The photographic image of sample C is also demonstrated in Fig. 7, whose transmittances is 89.06% at 550 nm (excluding PET substrate).

**Table 1:** Transmittances (excluding PET substrate) at 550 nm and sheet resistances of CuNW TCEs

Sample	A	B	C	D	E
*R*_*s*_ ($\Omega /\square $)	87.21	62.51	26.23	24.15	16.68
Transmittance	92.53%	90.77%	89.06%	81.46%	77.84%

### Fabrication of CuNW/PMMA HTCE

**Fig. 8:**
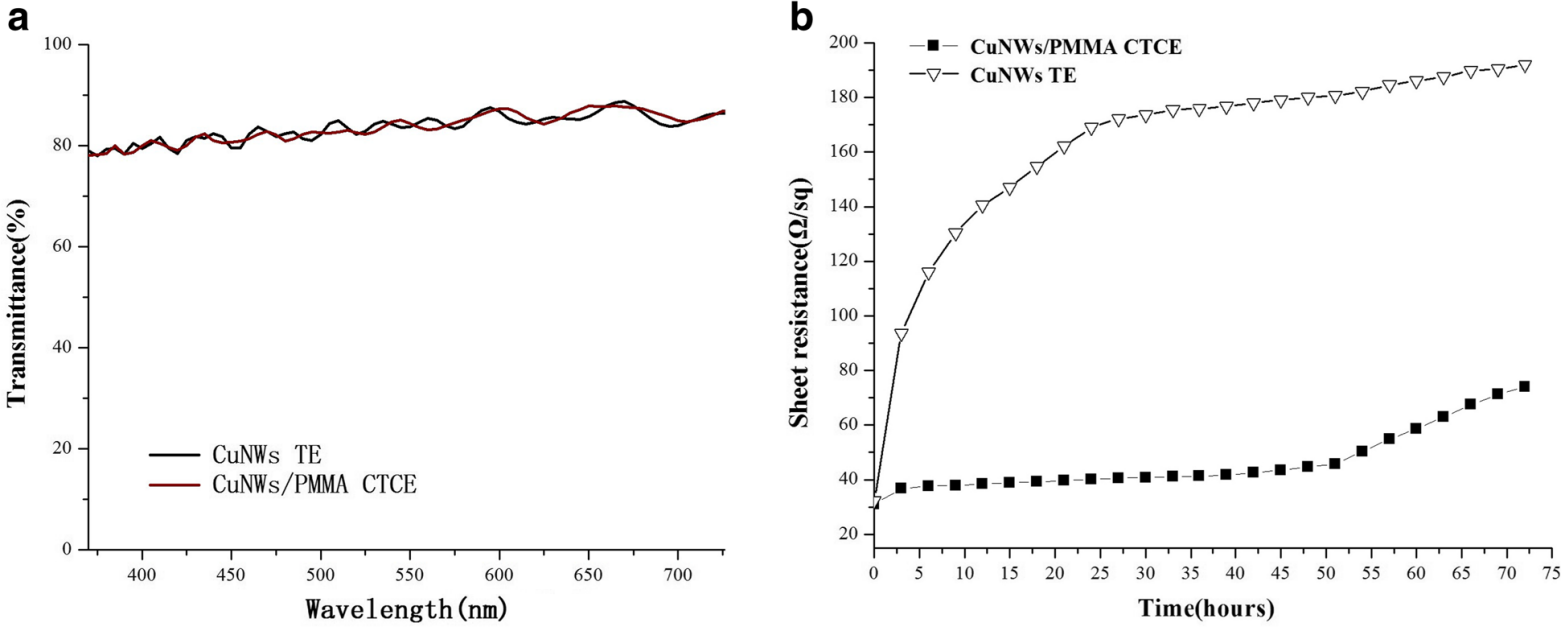
Optoelectronic properties comparison of a CuNW TCE and a CuNW/PMMA HTCE. **a** CuNW TCE and CuNW/PMMA HTCE possess similar transmittance spectra. **b** The change in the sheet resistance of CuNW and CuNW/PMMA HTCEs stored under ambient conditions for 72 h

Although CuNW TCEs have many advantages, some fatal shortcomings cannot be ignored, including high roughness and low chemical stability, which limit their applications. In order to overcome these problems, we fabricated CuNW/PMMA HTCEs by spin coating PMMA solution 20 mg/ml to reduce the roughness and prevent the CuNWs oxidation. The properties of the CuNW TCEs and CuNW/PMMA HTCEs were compared. Figure 8a, b shows transmittance graphs and the changes in conductivity of a CuNW TCE and a CuNW/PMMA HTCE on a PET substrate, respectively. Figure 8a shows that the CuNW TCE and the CuNW/PMMA HTCE possess similar transmittance spectra. From Fig. 8b, we can see that sheet resistances of the CuNW TCE and the CuNW/PMMA HTCE were also similar to each other when they have just been prepared. Sheet resistance of the CuNW TCE increased rapidly from 32.1 $\Omega /\square $ to 93.5 $\Omega /\square $ after 3 h. However, sheet resistance of CuNW/PMMA HTCE showed a slow increase, which was almost unchanged when 50 h passed and remained still very low (74 $\Omega /\square $) when 72 h passed. The stability was significantly improved after the CuNW TCE was coating with PMMA to form a CuNW/PMMA HTCE. Thus, the PMMA coating effectively protected the CuNWs from moisture and oxygen.

**Fig. 9:**
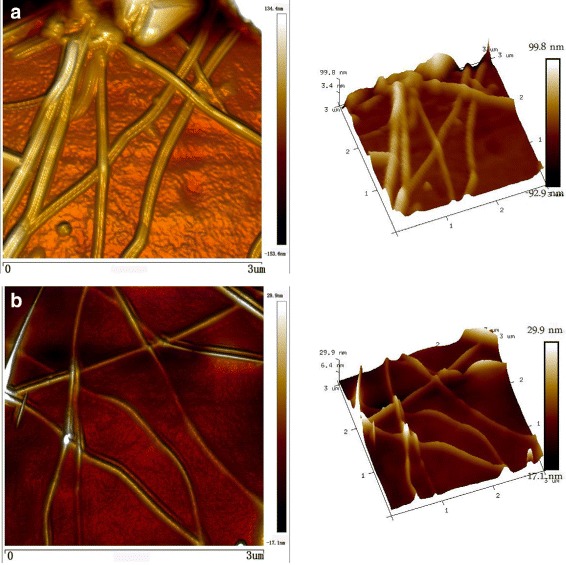
AFM topographic images of **a** CuNW TCE and **b** CuNW/PMMA HTCE. The left images are original AFM images, and the right images show the RMS surface roughness

As mentioned above, large roughness is not compatible with various applications and can cause short circuits in electronic devices. Therefore, smooth surfaces are crucial to the practical application of the optoelectronic devices. We embedded CuNWs in the PMMA film to reduce roughness. Figure 9 demonstrates AFM topographic images of a CuNW TCE and a CuNW/PMMA HTCE. In Fig. 9a, the surface topography of the CuNW TCE is relatively rough, whose root-mean-square (RMS) surface roughness is 31.2 nm. In Fig. 9b, the surface topography of the CuNW/PMMA HTCE appears very smooth, whose RMS surface roughness is 4.8 nm. It is obvious that the spin coated PMMA film can greatly reduce surface roughness of the CuNW film because the PMMA solution can fill holes between the random grids of CuNWs.

## Conclusions

A hydrothermal method for synthesizing ultralong and thin CuNWs is proposed in this paper. The average diameter of the CuNWs is about 35 nm, and the average length is about 100 *μ*m, and the corresponding aspect ratio is about 2857. The concerning raw materials include CuCl_2_·2H_2_O, ODA and ascorbic acid, which are all very cheap and nontoxic. In the hydrothermal process, CuCl_2_·2H_2_O provides copper source, ascorbic acid acts as the reducing agent, and ODA was used as the capping agent.

CuNW TCEs were fabricated by CuNWs prepared by the hydrothermal method. The best result we achieved for the CuNW TCEs was *R*_*s*_= 26.23 $\Omega /\square $ for T= 89.06% at 550 nm (excluding PET substrate). The TCE fabrication process was carried out at room temperature, and there was no need for post-treatment such as thermal annealing and opto-thermal heating. To reduce roughness and prevent against oxidation of CuNW TCEs, we fabricated CuNW/PMMA HTCEs. The experiments showed that CuNW/PMMA HTCE possessing lower roughness and higher antioxidant activity compared to CuNW TCE.

## Abbreviations

AFM: Atomic force microscope
AgNWs: Silver nanowires
CuNPs: Copper nanoparticles
CuNWs: Copper nanowires
HDA: Hexadecylamine
HRTEM: High resolution transmission electron microscopy
HTCE: Hybrid transparent conductive electrode
HTCEs: Hybrid transparent conductive electrodes
ITO: Indium Tin Oxide
MCE: Mixed cellulose ester
ODA: Octadecylamine
OLEDs: Organic light-emitting diodes
PET: Polyethylene terephthalate
PMMA: Poly(methyl methacrylate)
PVP: Polyvinyl pyrrolidone
RMS: Root-mean-square
TCE: Transparent conductive electrode
TCEs: Transparent conductive electrodes
TEM: Transmission electron microscopy
XRD: X-ray diffraction
